# Nitrogen remobilization and conservation, and underlying senescence‐associated gene expression in the perennial switchgrass *Panicum virgatum*

**DOI:** 10.1111/nph.13898

**Published:** 2016-03-03

**Authors:** Jiading Yang, Eric Worley, Qin Ma, Jun Li, Ivone Torres‐Jerez, Gaoyang Li, Patrick X. Zhao, Ying Xu, Yuhong Tang, Michael Udvardi

**Affiliations:** ^1^ Plant Biology Division the Samuel Roberts Noble Foundation Ardmore OK 73401 USA; ^2^ BioEnergy Sciences Center (BESC) Oak Ridge National Laboratory Oak Ridge TN 37831 USA; ^3^ Department of Plant Science South Dakota State University Brookings SD 57007 USA; ^4^ Department of Biochemistry and Molecular Biology University of Georgia Athens GA 30602 USA

**Keywords:** gene expression, nitrogen remobilization, senescence, switchgrass (*Panicum virgatum*), transcription factors

## Abstract

- Improving nitrogen (N) remobilization from aboveground to underground organs during yearly shoot senescence is an important goal for sustainable production of switchgrass (*Panicum virgatum*) as a biofuel crop. Little is known about the genetic control of senescence and N use efficiency in perennial grasses such as switchgrass, which limits our ability to improve the process.
- Switchgrass aboveground organs (leaves, stems and inflorescences) and underground organs (crowns and roots) were harvested every month over a 3‐yr period. Transcriptome analysis was performed to identify genes differentially expressed in various organs during development.
- Total N content in aboveground organs increased from spring until the end of summer, then decreased concomitant with senescence, while N content in underground organs exhibited an increase roughly matching the decrease in shoot N during fall. Hundreds of senescence‐associated genes were identified in leaves and stems. Functional grouping indicated that regulation of transcription and protein degradation play important roles in shoot senescence. Coexpression networks predict important roles for five switchgrass NAC (NAM, ATAF1,2, CUC2) transcription factors (TFs) and other TF family members in orchestrating metabolism of carbohydrates, N and lipids, protein modification/degradation, and transport processes during senescence.
- This study establishes a molecular basis for understanding and enhancing N remobilization and conservation in switchgrass.

## Introduction

Biofuel produced from lignocellulosic biomass of plants, particularly perennial grasses and trees, is a promising and sustainable alternative to fossil fuels (McLaughlin *et al*., 2002; Karp & Shield, 2008). It has been projected that 30% of current US petroleum consumption will be replaced with bioethanol by 2030. This will require billions of tons of biomass annually (Bouton, 2007). Switchgrass (*Panicum virgatum*), native to the North American tall grass prairies, is a perennial grass with water‐efficient C_4_ photosynthesis that was selected for development as a bio‐energy crop by the US Department of Energy. There are two switchgrass ecotype classes: ‘upland’ and ‘lowland’, which refer to latitude, not altitude. Average annual aboveground biomass yields of switchgrass in the USA were estimated to be 12.9 and 8.7 tons ha^−1^ for lowland and upland ecotypes, respectively (Wullschleger *et al*., 2010).

For a successful biofuel production system, high biomass yield and processing quality with minimal and sustainable inputs of water, fertilizers, and other chemicals are desirable (Karp & Shield, 2008). Increases in switchgrass biomass production potential have been achieved via classical breeding (Sanderson *et al*., 2006), while transgenic and plant–microbe symbiotic approaches show promise in this regard (Ghimire *et al*., 2009; Fu *et al*., 2012). Reduced recalcitrance of biomass to conversion into liquid fuels is a key quality trait, which is being pursued by reducing lignin content in switchgrass via transgenic approaches (Fu *et al*., 2011; Saathoff *et al*., 2011; Shen *et al*., 2012). High nutrient use efficiency, including nutrient remobilization to nonharvested organs, is an important target of sustainable biomass production in switchgrass.

Switchgrass plants can produce shoots > 3 m in height and develop a deep root system, which may extend to depths of > 3.5 m after several years of growth (Weaver, 1968). Nutrient remobilization during yearly shoot senescence of perennial grasses has been investigated intensively at the agronomic level because of its relationship to biomass yield and quality (Lemus *et al*., 2008; Heaton *et al*., 2009; Nassi o Di Nasso *et al*., 2011, 2013; Strullu *et al*., 2011; Dohleman *et al*., 2012; Kering *et al*., 2012). Remobilization of nutrients from senescing tissues and temporary storage in living underground organs presumably improve nutrient use efficiency and the ecological fitness of switchgrass (Bouton, 2007). Nonetheless, substantial amounts of soil nutrients are removed with harvested shoot biomass under cropping scenarios (Propheter & Staggenborg, 2010; Guretzky *et al*., 2011). For example, the total nitrogen (N) removed with biomass varied from 31 to 63 kg N ha^*−*1^ yr^*−*1^ in a one‐cut fall harvest system, and from 90 to 144 kg N ha^*−*1^ yr^*−*1^ for a two‐cut system, over 5 yr of measurements (Reynolds *et al*., 2000). Such nutrient withdrawal rates necessitate the addition of fertilizer N to maintain switchgrass productivity in the next growth season and will inevitably result in N depletion of soils without N supplementation. Therefore, decreasing the nutrient content in harvested switchgrass biomass, apart from structural and nonstructural carbohydrates that are converted to biofuels, by improving nutrient remobilization during yearly senescence will help to conserve soil nutrients, reduce the need for fertilizers, and reduce the economic and environmental costs of switchgrass biomass production. Successful development of switchgrass for biofuel production will depend, in part, on agricultural sustainability including nutrient conservation in the soil–plant system (Parrish & Fike, 2005; Yang *et al*., 2009).

Despite its importance for sustainable biomass production, we know very little at the physiological and molecular levels about N remobilization during switchgrass yearly senescence. Among the unknowns are the dynamics of nutrient accumulation then loss from shoot tissues and organs during growth and senescence, the molecular processes that are engaged during nutrient remobilization, and the genetic regulation of these processes. Knowledge of the latter is important if we hope to enhance nutrient use efficiency in switchgrass via directed genetic modifications. A variety of transcription factors (TFs) have been implicated in the regulation of senescence and N remobilization in the model plant *Arabidopsis thaliana* and other species, especially TFs of the NAC (NAM, ATAF1,2, CUC2) (Guo & Gan, 2006; Uauy *et al*., 2006; Kim *et al*., 2009; Balazadeh *et al*., 2011; Wu *et al*., 2012), WRKY (Robatzek & Somssich, 2001; Zentgraf *et al*., 2010; Zhou *et al*., 2011; Besseau *et al*., 2012) and MYB families (Guo & Gan, 2011; Jaradat *et al*., 2013). However, we are far from a comprehensive understanding of all TFs involved in senescence in any plant species, especially in perennial grass species such as switchgrass that are likely to form the backbone of the future biofuel industry.

In this study, we measured changes in N concentration and content in the various organs of switchgrass over three consecutive years. The results revealed major shifts of N between organs during growth and senescence of shoots. Transcriptome analysis of different organs at various developmental stages was carried out to identify genes, processes, and potential transcriptional regulators involved in N remobilization in switchgrass. The results of this work are presented here.

## Materials and Methods

### Plant material

A plastic‐covered hoop‐house open to the environment was used to grow switchgrass plants in this study, in preference to a field plot or a glasshouse, for two reasons. First, switchgrass (*Panicum virgatum* L.) plants grown in the field can develop enormous root systems with lengths of > 3.5 m (Weaver, 1968), which makes it almost impossible to harvest the entire root system. Growing plants in pots solved this problem, although pots can limit root growth. Second, switchgrass plants grown in our glasshouse do not undergo annual senescence of whole shoots; while some lower leaves senesce and turn yellow, most do not. By contrast, plants grown in our hoop‐house exhibited shoot senescence each year in the fall, like field‐grown plants (Supporting Information Fig. S1).

Seeds of switchgrass lowland cultivar ‘Alamo’ were germinated on sterile wet filter paper for 4–6 d and seedlings were then transferred into 1‐gallon pots containing a mixture of Metro‐Mix 350 (Sun Gro Horticulture, Agawam, MA, USA) and sand (12 : 1, v/v) in April 2008. Plants were grown in a semi‐open ‘hoop‐house’ with a transparent plastic sheet as a roof (Fig. S1). Thus, they were exposed to natural conditions of day‐length and temperature but with artificially controlled water and fertilizer supply. The hoop‐house is located on the campus of The Samuel Roberts Noble Foundation, Ardmore, Oklahoma (34°N, 97°W).

Plants were watered every week and fertilized with half‐strength Hoagland's solution (Hoagland & Arnon, 1950) every 2 wk at a rate of 200 ml per pot (from April to September 2008). One‐year‐old plants with similar aboveground biomass were selected and transferred into 3‐gallon pots in March 2009 for subsequent experiments. In addition to weekly watering, plants were fertilized with full‐strength Hoagland's solution (1 l per pot) once per month (the day after sample collection) from April to September. Once per month (around the 18th) from April 2009 to November 2011, three randomly selected plants were harvested in the morning (09:00–12:00 h) for destructive analyses.

### Sample harvesting

Switchgrass plants were cut as evenly as possible into two halves: one half for RNA extraction and one for N measurement. The aboveground material was separated into leaves, stems (including sheathes) and inflorescence (if present). Underground tissues were washed quickly with tap water to remove soil and separated into roots and crowns (including rhizomes). Samples for RNA extraction were frozen in liquid N, then stored at −80°C until further processing. Post‐senescent aboveground tissues harvested in October or later yielded poor‐quality or no RNA. Samples for nutrient measurements were dried at 65°C.

### Nitrogen measurement

Dried samples were ground into powder using an 6870 Freezer/Mill (SPEX SamplePrep, Metuchen, NJ, USA). The concentration of total N in each sample was analyzed by Ward Laboratories Inc. (Kearney, NE, USA) using a combustion method (Horneck & Miller, 1998) and expressed as mg N g^−1^ dry tissue. The N content was calculated by multiplying N concentration by total biomass (g).

### RNA extraction, microarray hybridization and gene selection

Samples selected for microarray analysis included leaves from June to September, stems from July to September, and crowns and roots from August to November, all harvested in 2009. Samples for leaves, stems and crowns included three biological replicates, while single samples were analyzed for roots. Total RNA was extracted from samples pulverized in liquid N, using a cetyltrimethylammonium bromide and LiCl method (Chang *et al*., 1993). RNA samples were treated with DNase I, evaluated for purity using a Bioanalyzer 2100 (Agilent; http://www.agilent.com), and then used for microarray analysis using Affymetrix cDNA microarray chips (Pvi_cDNAa520831), as described previously (Zhang *et al*., 2013). Probe labeling, array hybridization and scanning were performed by the Genomics/Microarray Facility at the Noble Foundation (Zhang *et al*., 2013).

Gene transcripts were classified as ‘detected’ in any organ if the average signal intensity at any single time‐point was ≥ 100. A gene was regarded as differentially expressed if its transcript ratio in any month relative to that of the first month was ≥ 2.0 or ≤ 0.5 at the *P *<* *0.1 level for leaves, stems and crowns with three biological replicates. For roots with one replicate, a three‐fold change in transcript ratio was set as the threshold.

### Senescence‐associated gene identification

Previously identified or predicted senescence‐associated genes (SAGs) from Arabidopsis, rice (*Oryza sativa*), wheat (*Triticum aestivum* and *Triticum turgidum*) and maize (*Zea mays*) collected in the Leaf Senescence Database (LSD) (Liu *et al*., 2011; Li *et al*., 2012) were used as references to identify putative conserved SAGs in switchgrass. tblastn was used to homology‐map each reference SAG to switchgrass unitranscripts and only those unitranscripts showing a blast e‐value < 1e‐30 and b‐score > 90% were kept. Resulting unitranscripts showing differential expression were further filtered based on increased expression during development in different organs, compared with the first month sample as a reference point, which yielded a list of putative switchgrass SAGs (PvSAGs).

### Functional grouping of AtSAGs

The program (Pathways: Overview) of mapman (Thimm *et al*., 2004) was used to classify gene function of AtSAGs homologous to PvSAGs. The mapping file used was Ath_AGI_LOCUS_TAIR10_Aug2012.m02. Sorted genes involved in regulation of transcription and protein degradation were used to find corresponding switchgrass senescence‐associated TF genes and protein degradation genes (PDGs). The classification of Arabidopsis TF families followed the *Arabidopsis thaliana* Transcription Factor Database (AtTFDB) housed on the Arabidopsis Gene Regulatory Information Server (AGRIS) (http://arabidopsis.med.ohio-state.edu/AtTFDB/) (Palaniswamy *et al*., 2006).

### Sequence alignment and phylogenetic analysis of NAC transcription factors

Coding sequences (CDSs) of NAC TF genes were determined using dnastar software (Dnastar Inc., Madison, WI, USA). For apparently incomplete coding sequences, blastn was performed using the original transcript sequences to identify longer, assembled ‘unique transcript sequences’ (UTs; http://switchgrassgenomics.noble.org). Putative complete CDSs were obtained by assembling the original transcript and blast‐recovered UTs, using Seq‐Man (dnastar). All putative NAC coding sequences were translated into protein sequences and then aligned using clustalw of megalign (dnastar) with the default parameter values. The alignment results were then used to produce a neighbor‐joining phylogenetic tree using mega5 (Tamura *et al*., 2011). The bootstrap method, with 100 replications, was used to provide confidence levels (reported as percentages) of branch points on the phylogenetic tree.

### Constructing a regulatory network around NAC transcription factors in shoots

To find specific PvSAGs with similar expression patterns to NAC genes potentially involved in the regulation of senescence and N remobilization, qubic software was used for biclustering analysis of PvSAG gene expression data obtained under multiple conditions (Li *et al*., 2009). SAGs predicted to be coexpressed with NAC genes were mapped to Arabidopsis via blastn with an e‐value < e‐30. The mapped Arabidopsis genes (excluding duplicates for various NACs) were then subjected to mapman (Pathway: Overview) analysis for functional grouping (Thimm *et al*., 2004). The role of some genes with unassigned function or BinName as miscellaneous (BinCode 26) was referred manually to The Arabidopsis Information Resource (TAIR) (https://www.arabidopsis.org). The corresponding PvSAGs were used to construct a putative regulatory network.

## Results

### Biomass accumulation in different organs

**Figure 1:**
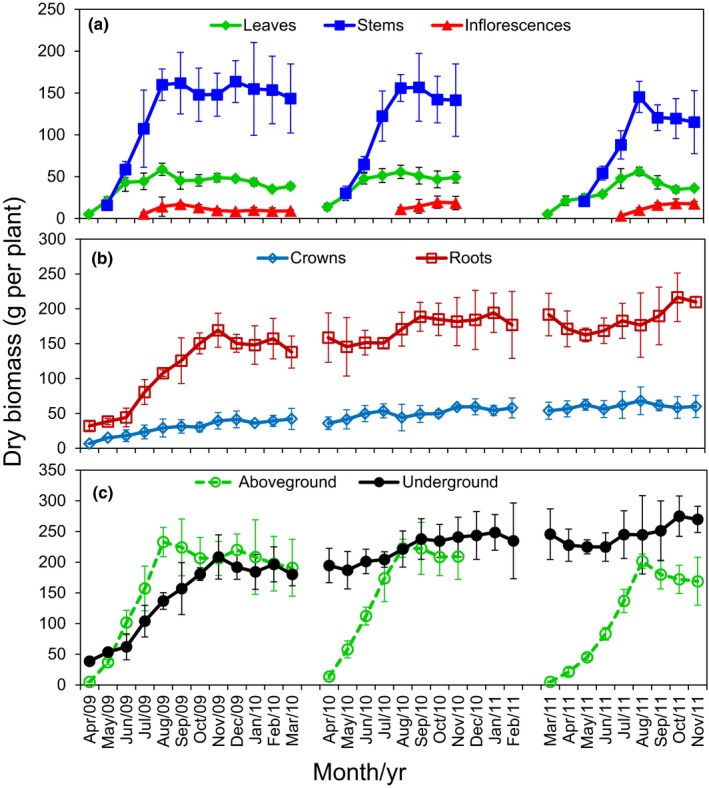
Biomass in switchgrass (*Panicum virgatum*) (a) aboveground organs (leaves, stems and inflorescences) and (b) underground organs (crowns and roots), and (c) calculated total biomass over time. The values are mean ± SD from three biological replicates.

Total aboveground biomass per plant was relatively low at *c*. 60 g DW in the year of establishment (2008) but increased approximately three‐fold to *c*. 185 g in subsequent years (Fig. S2). During each growth season following establishment in 2008, leaf and stem biomass increased during spring (March–May), after which leaf biomass plateaued in early summer (June) while stem biomass continued to increase until late summer (August) when it also plateaued (Fig. 1a). Inflorescence development occurred during summer and accounted for *c*. 5% of total biomass by the end of the year. Stems accounted for *c*. 75% and leaves *c*. 20% of total aboveground biomass at harvest after shoot senescence.

For underground organs, root biomass increased rapidly from April (32 g average) to November (169 g average) in 2009, then much more slowly through to November 2011, possibly as a result of spatial limitation imposed by the 3‐gallon pots. Root biomass accounted for *c*. 70–80% of total underground biomass over the experimental period. Crown biomass increased slightly in all three years and represented *c*. 20–30% of total underground biomass (Fig. 1b). Total aboveground (leaves, stems and inflorescences) and underground (roots and crowns) biomasses were similar at the end of the second and third growing seasons (2009 and 2010), while aboveground biomass was lower than underground biomass in the final year of the study (2011; Fig. 1c).

### Nitrogen concentration in different organs

**Figure 2:**
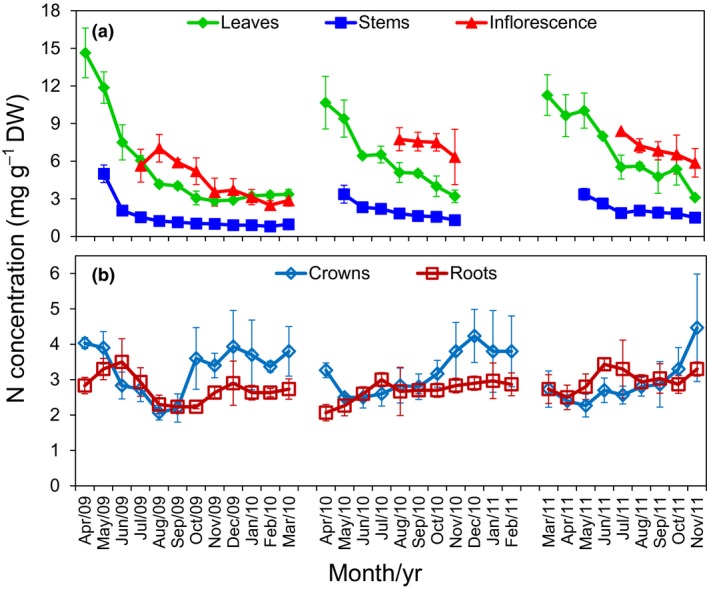
Changes of nitrogen (N) concentration in different switchgrass (*Panicum virgatum*) organs over time. (a) Nitrogen concentration in aboveground organs (leaves, stems and inflorescences). (b) Nitrogen concentration in underground organs (crowns and roots). The value at each time‐point indicates mean ± SD from three biological replicates.

N concentrations (mg g^−1^ DW) were highest in leaves and stems during the period of most rapid growth, in spring of each year (Fig. 2a). N concentrations in leaves and stems declined by *c*. 80% from the start of the growing season (March–April) to postsenescence (November). Leaves always had a higher N concentration (about three‐fold higher) than stems during both the vegetative and reproductive phases. When plants entered the reproductive stage (late July or early August), inflorescences had the highest N concentration of the three aerial organs (Fig. 2a). N concentration in roots increased in spring (from March–April to June–July), then decreased until September or October, and increased again following senescence of aboveground tissues (Fig. 2b). N concentration in crowns decreased during spring and showed a similar trend to roots in other months, although N concentration in the crown showed a larger fluctuation than in roots (Fig. 2b). Notably, our results revealed a period during which N concentration in the crown was higher than in roots (i.e. after September or October until next May or June) when plants were in winter dormancy and in early regrowth.

### Nitrogen content of different organs

**Figure 3:**
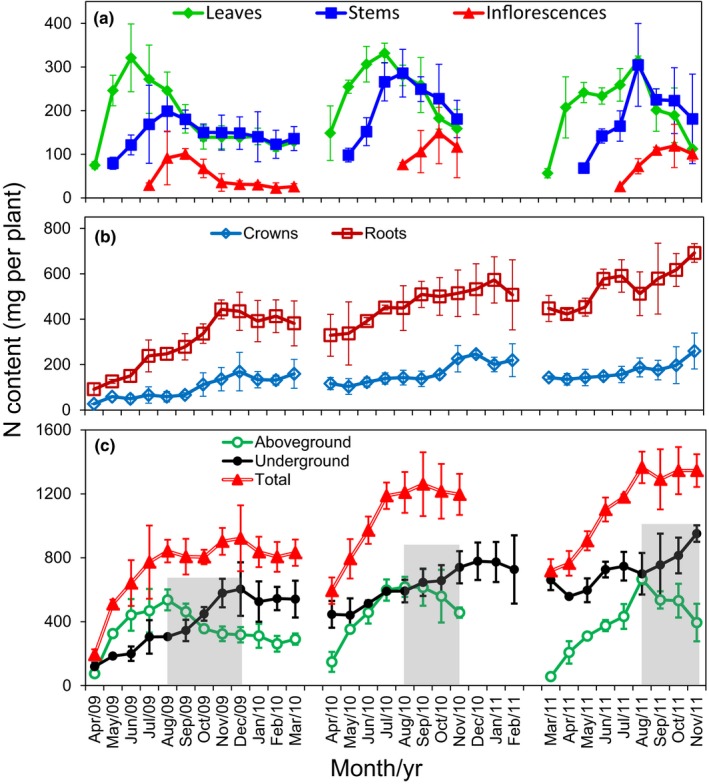
Changes of nitrogen (N) content in switchgrass (*Panicum virgatum*) over time. (a) N content in leaves, stems and inflorescences. (b) N content in crowns and roots. (c) Comparison of N content between aboveground and underground organs. Total N (sum of aboveground and underground N) per plant is also shown. The value at each time‐point is mean ± SD from three biological replicates. The shaded areas in (c) indicate periods during which N content in aboveground organs declined while that of underground organs increased.

N content, obtained by multiplying N concentration by biomass, in the three aboveground organs (leaves, stems and inflorescences) exhibited a similar pattern over the three growing seasons (2009–2011): N content first increased, reached a peak level, and then declined (Fig. 3a). Interestingly, different organs exhibited peak N in different months. In 2009, leaves showed peak N in June, and stems and inflorescences in September; in 2010, leaves showed peak N in July, stems in August, and inflorescences in October; while in 2011, leaves and stems showed peak N in August and inflorescences in October. In general, peak N occurred first in leaves, then in stems, and finally in inflorescences. Differences in the timing of peak N in different years presumably reflected variation in environmental conditions, such as ambient temperature, humidity, and irradiation, across the years. For underground organs, roots always had a higher N content than crowns (Fig. 3b). N contents of both roots and crowns gradually increased during summer and fall, concomitant with the gradual increase in biomass of these organs (Fig. 1).

### Nitrogen remobilization from aboveground to underground organs and nitrogen removal by aboveground harvesting

To investigate N translocation during annual senescence of the shoot, we calculated N contents of aboveground organs (sum of leaves, stems and inflorescences) and underground organs (sum of roots and crowns). Aboveground N content increased during spring, peaked in summer (August) and decreased during fall in all three years (Fig. 3c). By contrast, the N content of underground organs increased during spring and summer before leveling off in fall and declining slightly over winter and early spring (Fig. 3c). Interestingly, the loss of total N in the aboveground organs roughly matched the gain in N in the underground organs between summer and fall (August to November) in each of the three years (Fig. S3a), probably reflecting a source–sink relationship between the two sets of organs. Consistently, the total N content (sum of aboveground and underground N) in a growing plant increased during spring regrowth and reached a plateau after August (2009 and 2011) or July (2010) (Fig. 3c), reflecting conservation of total plant N during shoot senescence.

To estimate N remobilization efficiencies in aboveground organs during the three growing seasons, the N content postsenescence (in November or December) was compared with the N content peak value in each growing season; that is, N remobilization efficiency (NRE (%)) was calculated as (peak N content – postsenescence N content)/peak N content. For combined aboveground organs, N content peaked in August in all three years and NRE was 41% in 2009, 26% in 2010 and 43% in 2011. Typically, N was remobilized more effectively from leaves (NRE of 57%, 52% and 64% in 2009, 2010 and 2011, respectively) than from stems (NRE of 25%, 37% and 41% in 2009, 2010 and 2011, respectively; Fig. S3b). The total N removed with harvested aboveground biomass was 318, 456, and 394 mg per plant in 2009 (December), 2010 (November), and 2011 (November), respectively (Fig. S3c).

### Seasonal changes in gene expression in different organs

To identify genes and processes associated with N remobilization and conservation, gene transcript levels were measured in leaves from June to September, stems from July to September, and crowns and roots from August to November of 2009. Over these periods, N contents decreased in leaves and stems, and increased in crowns and roots (Fig. 3a–c). Using the first sampling month as a point of reference for each organ, a total of 13 034 genes showed differential expression (differentially expressed genes (DEGs); Table S1), with transcript levels increasing or decreasing at least two‐fold in at least one of the aerial organs and crowns, or three‐fold in roots over time. With overlaps among different organs, the number of DEGs was 5590 in leaves, 3769 in stems, 5462 in crowns and 4861 in roots (Table S1).

**Figure 4:**
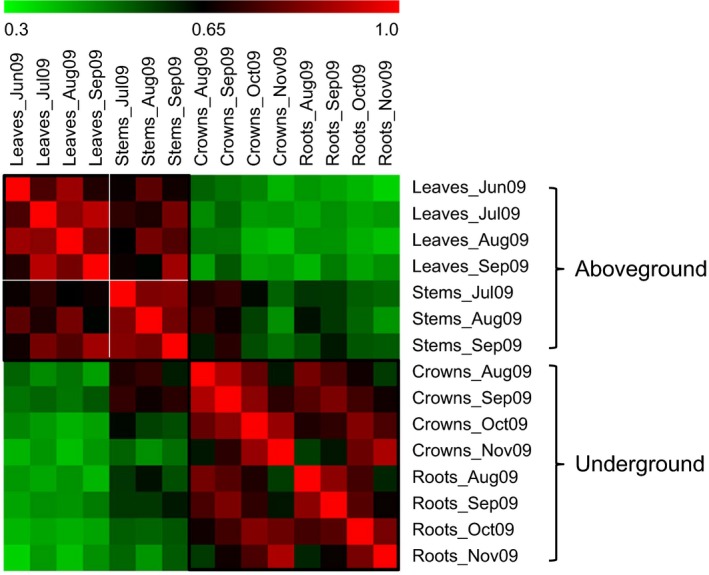
Comparisons among transcriptomes of various switchgrass (*Panicum virgatum*) organs. Pair‐wise Pearson correlation coefficients were calculated from transcript levels of all differentially expressed genes for all organs to generate the heat map. The color scale indicates the degree of correlation between transcriptomes of each pair of organs.

Pearson correlations of expression of these DEGs in different organs and/or at different time‐points revealed similarities in the transcriptomes of leaves and stems, and of crowns and roots, and dissimilarity between aboveground and underground organs (Fig. 4). For the aboveground group, the four leaf samples and three stem samples formed two separate subgroups, consistent with their distinct biological roles.

### Senescence‐associated genes in leaves and stems

Using reference SAGs from Arabidopsis, rice, maize and wheat, 597 putative switchgrass SAGs were identified in leaves and 437 SAGs in stems. All these SAGs showed an increase in expression greater than two‐fold during senescence (Table S2). Most of the switchgrass SAGs (PvSAGs; 583 in leaves and 428 in stems) had a corresponding Arabidopsis homolog (AtSAG), which aided classification of PvSAGs into functional groups using the mapman (Pathways: Overview) program.

The top two functional groups containing leaf or stem PvSAGs were RNA‐related processes (Bin 27; including RNA_processing, transcription, regulation of transcription, etc.) and protein metabolism (Bin 29; including protein synthesis, targeting, posttranslational modification, degradation, etc.; Table S3). Regulation of transcription (BinCode 27.3) was the largest subgroup in RNA processes, consisting of 87 leaf PvSAGs and 50 stem PvSAGs. Protein degradation (BinCode 29.5) was the largest subgroup in protein metabolism, with 39 leaf PvSAGs and 28 stem PvSAGs. These results highlighted the crucial role of transcriptional regulation and protein degradation in switchgrass shoot senescence and N remobilization. Other important functional categories encompassing PvSAGs included transport, development, signaling, hormone metabolism, stress, lipid metabolism and secondary metabolism (Table S3).

### Senescence‐associated genes involved in protein degradation in leaves and stems

Sequences of Arabidopsis SAG genes with functions in protein degradation (mapman BinCode 29.5) were used to identify 39 leaf and 28 stem senescence‐associated PDGs in switchgrass (Tables 1, S4). The majority of these PDGs encode components of ubiquitin‐dependent protein catabolic processes (BinCode 29.5.11), such as the ubiquitin‐activating enzyme (E1), ubiquitin‐conjugating enzyme (E2) and ubiquitin‐protein ligases (E3). Most of the E3 proteins were zinc finger C3HC4‐type RING family proteins and F‐box proteins of the Skp, cullin, F‐box (SCF) complex. Other PDGs encode proteins involved in the autophagy process or encode cysteine/aspartate/serine proteases, aminopeptidase, or AAA‐type ATPase family proteins (Table S4). Although all these PDGs exhibited at least two‐fold expression increases during leaf or stem development compared with a nonsenescent stage, their expression patterns were temporally distinct. For example, some leaf PDGs (AP13CTG21304, AP13CTG31154, AP13CTG01433, AP13CTG32069 and AP13CTG29791) showed expression peaks in August while others (e.g. AP13CTG02739, AP13CTG23243 and AP13CTG21021) exhibited a gradual increase in expression from June to September (Table S4). In view of the continuous decrease of total N in leaves from June to September (Fig. 3c), the varied temporal expression patterns of PDGs indicate distinct roles of different PDGs in protein recycling and N conservation at different developmental stages.

**Table 1:** Senescence‐associated protein degradation genes (PDGs) in switchgrass leaves and stems and their corresponding Arabidopsis homologs (see Supporting Information Table S4 for more details)

mapman BinCode	BinName	In leaves		In stems	
		PviUT sequence ID	Arabidopsis homolog	PviUT sequence ID	Arabidopsis homolog
29.5	protein.degradation	AP13CTG02178	AT1G23440	AlamCTG13082	AT4G33090
		AP13CTG23243	AT2G18600	AP13CTG42013	AT4G33090
		AlamCTG13082	AT4G33090		
		AP13CTG10608	AT4G33090		
		AP13CTG42013	AT4G33090		
29.5.11	protein.degradation.ubiquitin			AP13CTG03474	AT4G24690
29.5.11.1	protein.degradation.ubiquitin.ubiquitin	AP13ISTG55622	AT5G03240	AP13CTG13659	AT5G55160
29.5.11.2	protein.degradation.ubiquitin.E1			AP13CTG00940	AT5G06460
29.5.11.3	protein.degradation.ubiquitin.E2	AP13CTG04847	AT1G63800		
29.5.11.4.1	protein.degradation.ubiquitin.E3.HECT	AP13CTG29791	AT4G12570		
		AP13CTG19106	AT4G12570		
29.5.11.4.2	protein.degradation.ubiquitin.E3.RING	AP13CTG21304	AT1G02610	AP13CTG21304	AT1G02610
		AP13ISTG65978	AT1G08050	AP13ISTG73401	AT1G23030
		AP13ISTG73401	AT1G23030	AP13CTG07816	AT1G49850
		AP13CTG32069	AT3G07370	AP13CTG34502	AT2G15580
		AP13CTG31154	AT3G07370	AP13ISTG62924	AT2G20030
		AP13ISTG36348	AT3G47160	AP13ISTG68749	AT2G42360
		AP13CTG01797	AT5G10650	AP13CTG24769	AT3G16720
		AP13CTG10780	AT5G10650	AP13ISTG60909	AT3G46620
		AP13CTG18505	AT5G24870	AP13ISTG35994	AT4G35840
		AP13CTG09175	AT5G41350	AP13ISTG69037	AT5G01830
		AP13ISTG46430	AT2G30580	AP13ISTG32938	AT5G63970
		AP13CTG21021	AT1G73950	AP13CTG28718	AT5G63970
29.5.11.4.3.1	protein.degradation.ubiquitin.E3.SCF.SKP	AP13ISTG38065	AT5G59140		
29.5.11.4.3.2	protein.degradation.ubiquitin.E3.SCF.FBOX	AP13ISTG66687	AT1G68050	AP13CTG01225	AT1G14330
		AP13ISTG73845	AT1G80440	AP13CTG59148	AT1G21410
		AP13ISTG73847	AT1G80440	AP13CTG05982	AT1G21410
		AP13ISTG42605	AT2G03530	AP13ISTG35810	AT1G21410
29.5.11.4.5.2	protein.degradation.ubiquitin.E3.BTB/POZ Cullin3.BTB/POZ	AP13CTG02739	AT1G21780		
29.5.11.5	protein.degradation.ubiquitin.ubiquitin protease	AlamCTG14939	AT4G39910		
29.5.2	protein.degradation.autophagy	AP13ISTG52648	AT1G62040	AP13CTG05217	AT2G31260
		AP13CTG05792	AT3G07525		
		AP13CTG13363	AT3G15580		
		AP13CTG01433	AT5G54730		
29.5.3	protein.degradation.cysteine protease	AP13CTG12606	AT4G39090	AP13CTG20064	AT1G62710
		AP13CTG05252	AT4G39090	AP13ISTG71431	AT5G45890
		AP13ISTG73582	AT5G45890		
29.5.4	protein.degradation.aspartate protease	AP13CTG04573	AT1G11910	AP13CTG04573	AT1G11910
		AP13CTG11126	AT1G62290		
29.5.5	protein.degradation.serine protease	AP13CTG25528	AT5G53350	AP13ISTG67309	AT1G73270
				AP13CTG14014	AT5G38510
				AP13CTG05123	AT5G51070
29.5.9	protein.degradation.AAA type	AP13CTG19438	AT1G02890		

PviUT, switchgrass Panicum virgatum Unique Transcript.

### Senescence‐associated genes encoding transcription factors in leaves and stems

**Figure 5:**
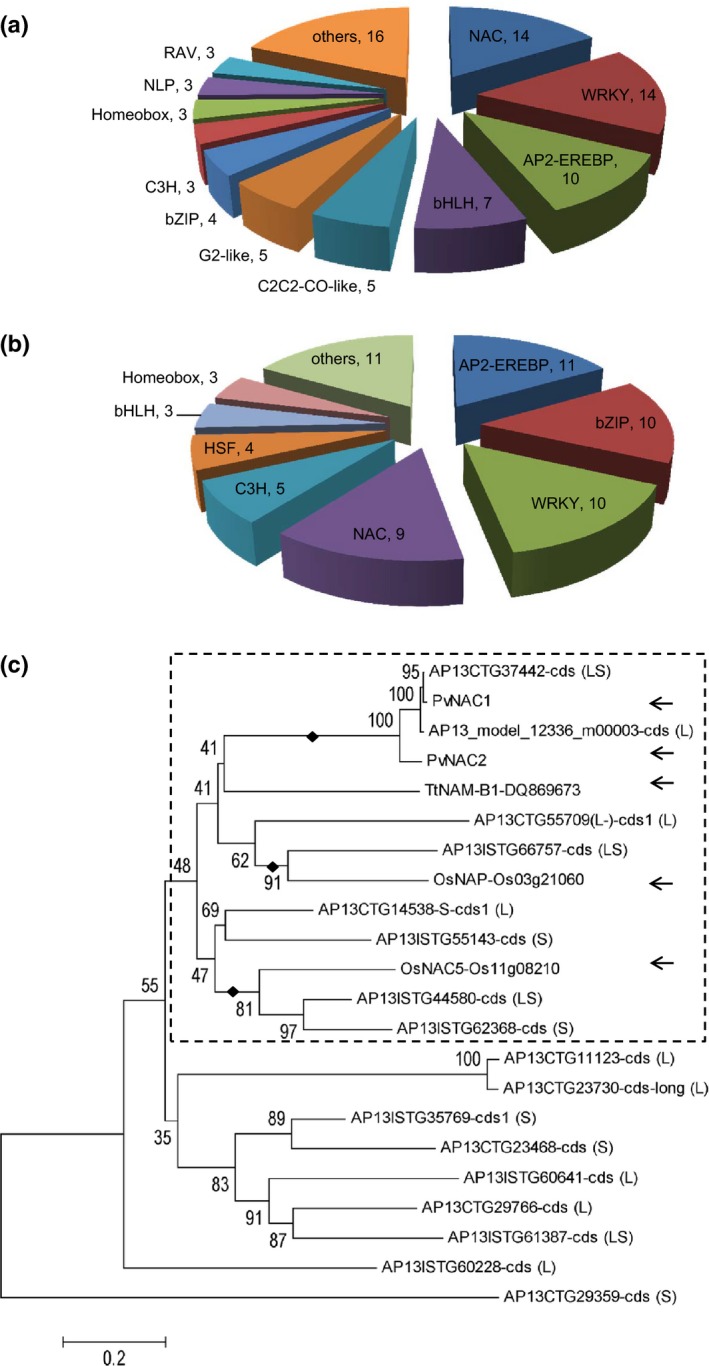
Senescence‐associated transcription factor (TF) genes in switchgrass (*Panicum virgatum*) leaves and stems. (a, b) Classification of TF families and respective gene numbers in (a) leaves and (b) stems. NAC, NAM, ATAF1,2, CUC2 family; AP2/EREBP, APETALA2/Ethylene Responsive Element Binding Protein family; bHLH, basic Helix‐Loop‐Helix family;C2C2‐CO‐like, C2C2‐type zinc finger‐CONSTANS‐like protein family; G2‐like, Golden 2‐like protein family; bZIP, basic‐leucine zipper family; C3H, CCCH‐type zinc finger protein family; NLP, NIN‐like protein family; RAV, Related to ABI3/VP1 family; HSF, Heat Shock Factor family. (c) Phylogenetic analysis of 17 NAC TFs in switchgrass leaves and stems. Five reference NAC proteins (TtNAM‐B1 (*Triticum turgidum* No Apical Meristem‐B1), OsNAC5, OsNAP (*Oryza sative* NAC‐like, Activated by AP3/PI), PvNAC1 and PvNAC2; indicated by black arrows), previously associated with nitrogen remobilization in wheat, rice and switchgrass, are included for comparison. The letters L and S in parentheses following sequence IDs stand for leaves and stems in which the gene transcript was mainly expressed. Eight NACs closely related to the five reference NACs are enclosed in the dashed rectangle. Three sub‐branches with high bootstrap values (> 80) containing the reference NACs are marked with black diamonds.

The AGRIS (Palaniswamy *et al*., 2006) was used to identify 87 leaf and 66 stem PvSAGs encoding TFs in switchgrass (Table S5). The PvSAG TFs were classified mainly into NAC, WRKY, AP2/EREBP (APETALA2/Ethylene Responsive Element Binding Protein), bZIP (basic‐leucine zipper), C3H (CCCH‐type zinc finger protein), bHLH (basic Helix‐Loop‐Helix domain protein), and C2C2‐CO‐like (C2C2‐type zinc finger‐CONSTANS‐like protein) families (Fig. 5a,b). These TF genes are hypothesized to play roles in the regulation of senescence and nutrient remobilization in switchgrass leaves and stems, with their up‐regulation coinciding with decreases in total N in leaves and stems (Fig. 3a). Nineteen SAG TF genes were found in both leaves and stems, indicating the presence of common regulatory pathways in these two organs.

We were especially interested in NAC TFs, as NAC genes are known to have roles in leaf senescence and/or N remobilization in Arabidopsis, wheat and rice (Guo & Gan, 2006; Uauy *et al*., 2006; Sperotto *et al*., 2009). Furthermore, two NAC genes, *PvNAC1* and *PvNAC2*, have been associated with leaf senescence in switchgrass (Yang *et al*., 2015). Interestingly, one gene (AP13CTG37442) among the leaf SAG NACs shares 99.5% nucleotide identity with *PvNAC1*. As *PvNAC1* is from switchgrass cultivar Summer and AP13CTG37442 is from cultivar Alamo, the high nucleotide identity indicates that these genes may be orthologous.

In total, 19 senescence‐associated NAC TF genes were identified in leaves or stems, four of which were common to both leaves and stems (Table S5). All but two of these gene sequences (AP13ISTG71006 and AP13ISTG72279) appear to encode the complete N‐terminal conserved domain that is typical of NAC family members (Ooka *et al*., 2003). Phylogenetic analysis of the 17 complete protein sequences was performed, together with previously characterized TtNAM‐B1 (*Triticum turgidum* No Apical Meristem‐B1), OsNAC5, OsNAP (*Oryza sative* NAC‐like, Activated by AP3/PI), PvNAC1 and PvNAC2 as reference sequences. Eight of the PvNAC SAGs are in the same clade as the five reference proteins (Fig. 5c). Five of these NACs (AP13CTG37442, AP13_model_12336_m00003, AP13ISTG66757, AP13ISTG44580 and AP13IST G62368) are particularly closely related to the five reference NACs, with relatively high bootstrap values (> 80) (Baldauf, 2003). Thus, these five NACs, which we subsequently call target NACs, are most likely to regulate transcription of genes involved in senescence and N remobilization in switchgrass shoots.

### Constructing a coexpression network around the five target senescence‐associated NAC transcription factors

Through bi‐clustering analysis using our in‐house program qubic (Li *et al*., 2009), 10 clusters were identified for PvSAGs in leaves and stems separately (Table S6). For leaf clusters, cluster 2 was found to contain three of the five target NAC genes mentioned earlier, namely AP13CTG37442, AP13_model_12336_m00003, and AP13ISTG66757. No leaf cluster contained the other two NACs (AP13ISTG44580 and AP13ISTG62368). For stem clusters, cluster 2 contained two target NAC members (AP13CTG37442 and AP13ISTG66757) and cluster 9 contained another two target NACs (AP13ISTG44580 and AP13ISTG62368). No cluster contained AP13_model_12336_m00003, which is mainly expressed in leaves (Fig. 5c).

**Figure 6:**
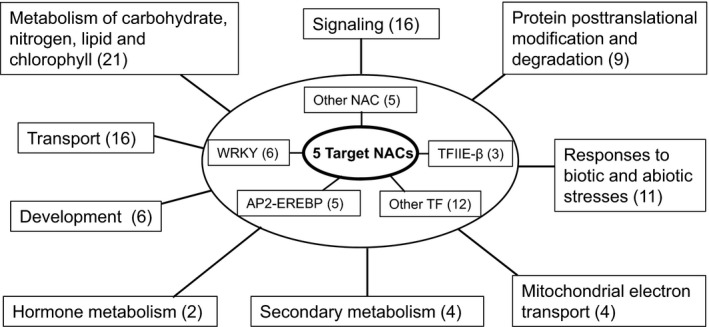
Network of 120 switchgrass (*Panicum virgatum*) senescence‐associated genes (PvSAGs) coexpressed with five target PvNACs. Transcription factors (within rectangles inside the larger oval) are potentially involved in the regulation of genes in the other functional groups, together with the five target NACs. NAC, NAM, ATAF1,2, CUC2 family; AP2/EREBP, APETALA2/Ethylene Responsive Element Binding Protein family; TFIIE‐β, Transcription Initiation Factor TFIIE, beta subunit. Numbers in parentheses indicate the number of genes in each group.

A total of 165 PvSAGs were co‐expressed with the five target NACs, and these matched 147 AtSAGs (Table S2). Based on functional annotations obtained from mapman (Pathways: Overview) and TAIR for the AtSAGs (75 unique loci), a coexpression network for switchgrass shoots was constructed around the five target NACs (Fig. 6). A total of 120 PvSAGs (corresponding to 60 unique and functionally annotated AtSAGs) coexpressed with the five target NACs were classified into 10 functional groups (Fig. 6; Table S7). The largest group was TFs which included 31 members mainly belonging to the NAC, WRKY, and AP2‐EREBP families (Table 2). The five target PvNACs together with another five NACs made this the largest TF subgroup. Prominent functional groups were metabolism of carbohydrate, N, lipid and chlorophyll (with 21 PvSAGs), transport (16 PvSAGs), signaling (16 PvSAGs), stress responses (11 PvSAGs), protein posttranslational modification and degradation (nine PvSAGs), development (six PvSAGs), and secondary metabolism (four PvSAGs). These functional groups cover most, if not all physiological processes known to be involved in leaf senescence (Lim *et al*., 2007), highlighting the crucial importance of transcriptional regulation in coordinating senescence‐related processes.

**Table 2:** Main transcription factor (TF) genes among switchgrass (*Panicum virgatum*) senescence‐associated genes (PvSAGs) that were coexpressed with the five target PvNACs (switchgrass NAM, ATAF1,2, CUC2 TFs)

Transcription factor subgroups	PvSAGs	Corresponding AtSAGs	Biological process or function of AtSAGs
**NAC**	**AP13_model _12336.m00003**	AT1G69490	ANAC029/NAP, regulation of transcription
	**AP13CTG37442**	AT1G69490	ANAC029/NAP, regulation of transcription
	**AP13ISTG66757**	AT1G69490	ANAC029/NAP, regulation of transcription
	**AP13ISTG44580**	AT1G01720	ANAC2/ATAF1, regulation of transcription
	**AP13ISTG62368**	AT1G01720	ANAC2/ATAF1, regulation of transcription
	AP13CTG29359	AT1G62700	ANAC026/VND5, regulation of transcription
	AP13ISTG71006	AT3G04070	ANAC047/SHG, regulation of transcription
	AP13CTG55709	AT3G04070	ANAC047/SHG, regulation of transcription
	AP13ISTG54654	AT3G12977	NAC family TF, regulation of transcription
	AP13ISTG54652	AT3G12977	NAC family TF, regulation of transcription
**WRKY**	AP13ISTG70516	AT3G56400	WRKY70, regulation of transcription
	AP13CTG27420	AT3G56400	WRKY70, regulation of transcription
	AP13ISTG43971	AT3G56400	WRKY70, regulation of transcription
	AP13ISTG43830	AT2G38470	WRKY33, regulation of transcription
	AP13ISTG33944	AT2G38470	WRKY33, regulation of transcription
	AP13ISTG73773	AT5G15130	WRKY72, regulation of transcription
**AP2‐EREBP**	AP13ISTG55667	AT4G17500	ERF1, regulation of transcription
	AP13ISTG73632	AT4G17500	ERF1, regulation of transcription
	AP13ISTG66196	AT4G17500	ERF1, regulation of transcription
	AP13CTG58854	AT4G17500	ERF1, regulation of transcription
	AP13CTG23914	AT4G17500	ERF1, regulation of transcription
**TFIIE‐β**	AP13ISTG49795	AT4G20330	Transcription initiation factor TFIIE, beta subunit; regulation of transcription
	AP13CTG09380	AT4G20330	Transcription initiation factor TFIIE, beta subunit; regulation of transcription
	AP13CTG22451	AT4G20330	Transcription initiation factor TFIIE, beta subunit; regulation of transcription

The PvNACs in bold are the five target NACs of most interest identified in Fig. 5(c). NAC, NAM, ATAF1,2, CUC2; AP2/EREBP, APETALA2/Ethylene Responsive Element Binding Protein; NAP, NAC‐like, Activated by AP3/PI; ATAF1, *Arabidopsis thaliana* Activating Factor 1; VND5, Vascular‐related NAC‐Domain protein 5; SHG, Speedy Hyponastic Growth; ERF1, Ethylene Response Factor 1.

### Genes induced in crowns and roots during remobilization of aboveground nitrogen

To gain deeper insight into genes potentially involved in N conservation in crowns and roots during shoot senescence (August–November), 3004 genes in crowns and 2451 in roots were identified as up‐regulated from the lists of DEGs (Table S1). These genes exhibited increased expression in at least 1 month following August (Table S8), during which period the aboveground N content decreased gradually (Fig. 3c).

Most of the genes induced in crowns (2402 of 3004 genes) and roots (1692 of 2451 genes) during shoot senescence have Arabidopsis homologs, which facilitated functional annotation and analysis. Broad functional analysis was performed using the mapman program (Pathways: Overview; Table S8). Many of the genes induced in crowns and roots fell into the protein‐related group (BinCode 29; 440 and 226 genes, respectively) or the RNA‐related processes group (263 and 170 genes, respectively), possibly indicating higher protein metabolism in these organs from August to November. Consistent with this idea, protein synthesis and targeting genes were highly represented among crown and root genes induced during shoot senescence (Table S8). More precisely, 193 of the 440 genes induced in crowns are linked to protein synthesis (BinCode 29.2), possibly for protein and N storage (Fig. 3c).

## Discussion

### Biomass production, nitrogen investments, and nitrogen conservation

Aboveground biomass production of switchgrass is the most important trait for the bioenergy industry. Each year, plant investments in biomass were first made in leaves and stems, during spring and summer, to support photosynthesis and further growth of vegetative organs and, later, the reproductive organs (Fig. 1). Photosynthesis also sustained root and crown growth throughout spring, summer, and fall. At the end of the growth period each year, stems accounted for *c*. 75% of dry shoot biomass, while leaves and inflorescences accounted for *c*. 20% and 5%, respectively (Fig. 1).

Major investments of N in spring were first made in the leaves, the most important photosynthetic organ. Smaller investments of N were made initially in stems for support of leaves, although total N content in stems eventually matched that in leaves (Fig. 3a). The N concentration in shoots was highest in young, developing organs, and declined as organs expanded to their mature size, and again as they senesced (Fig. 2). N concentration was always approximately three times higher in leaves than in stems, which presumably reflected higher metabolic activity in leaves, especially photosynthesis. Likewise, relatively high concentrations of N in inflorescences probably reflected high metabolic activity in this organ as it developed. N concentration in roots was more or less constant over time, and similar to that of growing stems (Fig. 2). However, total N in roots and crowns increased as these organs accumulated biomass (Fig. 3b). Total shoot N decreased during senescence, presumably as a means of conserving N elsewhere in the plant, especially in underground organs (Schwartz & Amasino, 2013). Consistent with this idea, the total N content in the root and crown increased by a similar amount during shoot senescence (Figs 3c, S3a). Furthermore, the subsequent decrease in the N content of roots and crowns in the following spring coincided with a rapid increase in N in the shoot organs in the same period (Fig. 3). Significant declines in crown N concentration during spring are consistent with this organ being a transient store of N for the rest of the plant (Cyr & Bewley, 1989; Volenec *et al*., 1996).

In each of the three years of this study, aboveground biomass of switchgrass reached a plateau in the summer (August; Fig. 1). Therefore, harvesting at any time after August would have maximized biomass yield. In contrast, the macronutrient content of shoots, including N, P and K, declined during shoot senescence (Fig. 3; Yang *et al*., 2009). Therefore, to maximize yield and minimize loss of valuable nutrients from the plant–soil system, harvesting after senescence is ideal. In fact, it has been estimated that up to 26.5 kg N ha^−1^ can be conserved in the root–soil system when switchgrass shoots are harvested after senescence (Wayman *et al*., 2014).

Switchgrass plants have multiple tillers that are usually at different developmental stages (Hardin *et al*., 2013). In grasses, the lower leaves on a tiller are physiologically older than those at the top (Sadras, 2000). Likewise, different parts of the root system vary in age and functional status (Santos *et al*., 2002). To avoid sample bias by collecting a small part of a specific organ, we harvested whole plants and pooled all organs of the same type, regardless of their developmental stage. The trade‐off with this approach is that it obscures developmental differences between organs of the same type. Nonetheless, this approach proved to be suitable for investigating seasonal redistribution of N between aboveground and underground organs (Fig. 3c) and for uncovering many of the accompanying gene expression changes (Fig. 4).

### Gene expression dynamics during switchgrass senescence

Genome‐wide studies of transcription in switchgrass are in their infancy (Li *et al*., 2013b; Zhang *et al*., 2013), with only one previous study of transcriptome changes underlying development, which focused on flag leaves (Palmer *et al*., 2015). In the present study, switchgrass Affymetrix chip analysis was conducted to identify global changes in gene expression in leaves, stems, crowns and roots related to seasonal shoot senescence and N remobilization from aboveground to underground organs (Fig. 3c). A total of 13 034 genes were found to be differentially expressed, either up‐ or down‐regulated with at least a two‐fold change in leaves, stems or crowns, or a three‐fold change in roots, during a time‐series spanning shoot maturation and senescence. Hierarchical clustering analysis (HCA), based on Pearson correlations of gene expression among different organs, revealed two main groups of transcriptomes (Fig. 4), indicating that aboveground organs (i.e. leaves and stems) and underground organs (i.e. crowns and roots) had distinctive gene expression patterns. Furthermore, in the aboveground group, four leaf samples and three stem samples constituted two separate subgroups. By contrast, differences between crowns and roots were less apparent, indicating greater overlap in development and differentiation of roots and crowns.

In recent years, genetic and genomic studies have revealed many of the molecular processes and some of the regulatory genes behind leaf senescence. In particular, transcriptome analyses of Arabidopsis revealed thousands of SAGs that are up‐regulated during normal, developmentally programed or stress‐induced senescence in Arabidopsis (Buchanan‐Wollaston *et al*., 2003; Lin & Wu, 2004; van der Graaff *et al*., 2006; Breeze *et al*., 2011). Loss of function of some SAGs results in delayed leaf or whole‐plant senescence and altered nutrient translocation (Uauy *et al*., 2006; Lim *et al*., 2007). An LSD has been constructed with fairly comprehensive information about SAGs, mutants, and phenotypes in different plant species (Liu *et al*., 2011; Li *et al*., 2014).

In this study, we focused on evolutionarily conserved SAGs in leaves and stems of switchgrass as these two organs are the main source of biomass for bioenergy production (Fig. 1) and the main source of macronutrients such as N and phosphorus (P) that are translocated to the root system during senescence for reuse during the next growth cycle. We used SAGs in Arabidopsis, rice, maize and wheat listed by LSD (Li *et al*., 2014) to identify related genes in switchgrass. Hundreds of switchgrass SAG homologs that exhibited increased expression during senescence were identified as *bona fide* PvSAGs (Table S2). mapman analysis of the corresponding well‐annotated AtSAGs revealed that RNA‐related processes, including RNA processing, transcription, and regulation of transcription, and protein metabolism, including protein synthesis, targeting, posttranslational modification, and degradation, were major processes involving PvSAGs (Table S3). Regulation of transcription and protein degradation were the top processes in these two functional categories, respectively. Other processes, such as transport, development, signaling, hormone metabolism, stress, lipid metabolism and secondary metabolism, were also identified as PvSAG‐encoded in leaves and stems, as was found in Arabidopsis (Guo *et al*., 2004; Buchanan‐Wollaston *et al*., 2005; van der Graaff *et al*., 2006).

### Genes involved in protein degradation

During plant senescence, various pathways may contribute to degradation of proteins and other macromolecules (van der Graaff *et al*., 2006). One highly conserved mechanism in eukaryotes is the ubiquitin‐proteasome pathway (Hellmann & Estelle, 2002), which involves covalent attachment of ubiquitin to protein substrates through the sequential action of three enzymes called a ubiquitin‐activating enzyme (E1), a ubiquitin‐conjugating enzyme (E2), and a ubiquitin‐protein ligase (E3). Ubiquitinated proteins are targeted for degradation by the 26*S* proteasome (Smalle & Vierstra, 2004). A number of genes involved in ubiquitination pathways are up‐regulated during leaf senescence in Arabidopsis, such as those encoding zinc finger (C3HC4‐type RING) proteins and other members of the ubiquitin ligase complex (Buchanan‐Wollaston *et al*., 2005; Breeze *et al*., 2011). In the present study, 23 of 39 protein degradation PvSAG genes in leaves were related to ubiquitin pathway genes, of which 12 encode zinc finger (C3HC4‐type RING finger) family proteins. In stems, 12 of the 19 ubiquitination‐related genes were C3HC4‐type RING finger proteins (Tables 1, S4). These results indicate that protein degradation via the ubiquitination pathway probably plays an important role in controlled protein degradation during shoot senescence in switchgrass. A variety of general proteases have been implicated in N recycling during plant senescence (Buchanan‐Wollaston *et al*., 2005; Roberts *et al*., 2012). We identified several SAG genes encoding cysteine, serine, or aspartate proteases in both switchgrass leaves and stems (Table S4). In wheat, serine proteases were reported to be responsible for much of the protein degradation that occurs during senescence of flag leaves, which drives N remobilization during grain filling (Chauhan *et al*., 2009; Roberts *et al*., 2011). Inhibition of cysteine proteases with inhibitors in wheat and tobacco (*Nicotiana tabacum*) leaves resulted in an impaired Rubisco degradation, indicating the involvement of cysteine proteases in degradation of plastid proteins (Thoenen *et al*., 2007; Prins *et al*., 2008). We also identified autophagy genes, four in leaves and one in stems, among switchgrass PDGs (Table S4). Accumulating evidence indicates that autophagy might manage the turnover of long‐lived proteins or damaged organelles (Avila‐Ospina *et al*., 2014). Collectively, our results indicate that multiple protein degradation pathways participate in N remobilization from switchgrass aboveground to underground organs during annual senescence (Fig. 3). Given the dearth of information about the mechanisms of N remobilization in perennial grasses, our results provide important and novel insight into the genes and proteins involved in protein catabolism during leaf and stem senescence in switchgrass, a useful model for other perennial grasses.

### Transcription factors regulating nitrogen remobilization during shoot senescence

Senescence is a highly regulated process, involving up‐ and down‐regulation of many genes (Buchanan‐Wollaston *et al*., 2003), by which the nutrients accumulated in aging tissues are remobilized to growing vegetative or reproductive organs (Lim *et al*., 2007). Senescence‐associated TFs play important roles in orchestrating controlled shoot senescence and effective N remobilization. We found 87 PvSAG TFs in switchgrass leaves and 66 in stems (Fig. 5a,b), mainly from the NAC, WRKY, AP2/EREBP, bZIP, C3H, bHLH and C2C2‐CO‐like families. Similar SAG TFs have been found in Arabidopsis (Guo *et al*., 2004; Buchanan‐Wollaston *et al*., 2005; van der Graaff *et al*., 2006; Guo, 2013), indicating that mechanisms of transcriptional control of senescence are likely to be conserved among dicots and monocots and in annual and perennial plant species.

NAC TFs constitute one of the largest families of plant‐specific TFs, and these proteins regulate a wide range of developmental processes in Arabidopsis, including seed development, shoot apical meristem formation, fiber development, and cell division (Ooka *et al*., 2003; Fang *et al*., 2008; Nuruzzaman *et al*., 2013). NAC TFs are known to regulate leaf senescence (Guo & Gan, 2006; Kim *et al*., 2009; Wu *et al*., 2012) and several NAC genes have been associated with N remobilization in wheat (Uauy *et al*., 2006), rice (Sperotto *et al*., 2009; Liang *et al*., 2014) and switchgrass (Yang *et al*., 2015). In the present study, NAC genes were among the most abundant groups of SAG TF genes found in switchgrass (Fig. 5a,b). Phylogenetic analysis of 17 SAG NAC TFs from leaves and stems revealed that five of them are closely related to NACs of other monocots that are known to be associated with N remobilization (Fig. 5c), indicating that these five switchgrass genes may potentially regulate N remobilization during senescence of switchgrass aboveground organs. They are clear targets for future work aimed at improving N remobilization efficiency in switchgrass.

Coexpression networks use correlation (or related measures) of gene expression profiles across multiple samples to group common biological functions or cellular processes (Eisen *et al*., 1998). We used qubic software as a biclustering analysis tool (Li *et al*., 2009) to find PvSAGs that are connected to five target NACs of interest (Fig. 5c). This method has been used to explore plant cell wall‐related genes in switchgrass (Chen *et al*., 2015). A total of 120 PvSAGs coexpressed with the five target PvNACs were classified into 10 functional groups (Table S7). Among the largest TF group, with 31 genes, there were five NACs in addition to the five target NACs. Six PvWRKY genes are homologous to *AtWRKY70*,* 33* and *72*, all of which have been reported to be involved in biotic responses (Bhattarai *et al*., 2010; Birkenbihl *et al*., 2012; Li *et al*., 2013a). *WRKY70* has also been found to act, together with *WRKY54,* as a negative regulator of leaf senescence in Arabidopsis (Besseau *et al*., 2012). It would be interesting to determine whether any of the PvWRKY TFs antagonize the function of PvNAC TFs in cells before or during senescence. All five AP2‐EREBP TF members of the PvNAC network are homologous to *AtERF1*, an ethylene response factor gene (Solano *et al*., 1998). Interestingly, another two genes in the network are homologous to *AtACO4*, encoding 1‐aminocyclopropane‐1‐carboxylate oxidase 4, an ethylene‐forming enzyme. These results indicate that ethylene biosynthesis and signaling may be involved in switchgrass shoot senescence, as is the case in Arabidopsis and other plants (John *et al*., 1995; Kim *et al*., 2009; Li *et al*., 2013c). Notably, nine genes coexpressed with target NACs fall into the functional group protein posttranslational modification and degradation, while 21 genes are associated with metabolism of N compound, starch/carbohydrate, lipid/fatty acid and chlorophyll. Sixteen genes probably encode transporters of carbohydrates, peptides, organic acids or metal ions (Table S7). Taken together, these results indicate that NACs and coexpressed TFs of other families together control the transcription of a wide range of genes involved in switchgrass shoot senescence and N remobilization. This developmental process also appears to involve complex signaling pathways, including calcium/calmodulin kinases, receptors, phosphorelays, and hormones (Table S7).

In summary, this study has revealed much of the molecular detail behind seasonal senescence of shoot organs in switchgrass and the associated remobilization and conservation of N in this perennial species. Key processes activated before and/or during senescence included transcriptional regulation of gene expression and protein degradation. NAC and other families of TFs emerged as likely regulators of senescence and nutrient remobilization in switchgrass, and these represent attractive targets for further study and are of potential use to enhance nutrient use efficiency in this target biofuel species.

## Author contributions

J.Y. and M.U. designed the research, analyzed the data and wrote the manuscript. J.Y. and E.W. grew plants and harvested samples. J.Y., E.W. and I.T‐J. performed experiments. Q.M., J.L., G.L., P.X.Z., Y.X and Y.T participated in the microarray and bioinformatics data analysis.

## Supporting information

Please note: Wiley Blackwell are not responsible for the content or functionality of any supporting information supplied by the authors. Any queries (other than missing material) should be directed to the *New Phytologist* Central Office.

**Fig. S1** Switchgrass plants grown in a hoop‐house.
**Fig. S2** Aboveground biomass in three successive years following switchgrass plant establishment in 2008.
**Fig. S3** Nitrogen remobilization between switchgrass aboveground and underground organs during yearly senescence.

**Table S1** Average expression of switchgrass differentially expressed genes, with two‐fold transcript change in leaves, stems and crowns (*n *=* *3), and with three‐fold change in roots (*n *=* *1)

**Table S2** List of senescence‐associated genes in switchgrass leaves and stems and their homologs, if available, in Arabidopsis, rice, maize and wheat

**Table S3** Functional groups of switchgrass leaf and stem PvSAGs classified by mapman (Pathways: Overview) for 426 and 321 homologous Arabidopsis loci, respectively

**Table S4** Expression of senescence‐associated protein degradation genes (PDGs) in switchgrass leaves and stems

**Table S5** List of PvSAGs encoding putative TFs in switchgrass leaves and stems and their homologs in Arabidopsis

**Table S6** Coexpression clusters of PvSAGs in switchgrass leaves and stems

**Table S7** The functional groups of switchgrass PvSAGs coexpressed with five target PvNACs

**Table S8** Expression of up‐regulated genes, their Arabidopsis homologs and functional grouping in switchgrass crowns and roots during remobilization of aboveground nitrogen
