# Supplementary material for: Nitrogen remobilization and conservation, and underlying senescence‐associated gene expression in the perennial switchgrass Panicum virgatum
Source: New Phytol. 2016 Mar 3;211(1):75–89. doi: 10.1111/nph.13898 (PMC6680227; doi:10.1111/nph.13898)
Supplement: Supplementary file 1 — Fig. S1 Switchgrass plants grown in a hoop‐house. Fig. S2 Aboveground biomass in three successive years following switchgrass plant establishment in 2008. Fig. S3 Nitrogen remobilization between switchgrass aboveground and underground organs during yearly senescence. [file NPH-211-75-s001.pdf]

New Phytologist

***New Phytologist* Supporting Information Figs S1–S3**

Article title: Nitrogen remobilization and conservation, and underlying senescence-associated gene expression in the perennial switchgrass, *Panicum virgatum*

Authors: Jiading Yang, Eric Worley, Qin Ma, Jun Li, Ivone Torres-Jerez, Gaoyang Li, Patrick X. Zhao, Ying Xu, Yuhong Tang and Michael Udvardi

Article acceptance date: 14 January 2016

The following Supporting Information is available for this article:

Fig. S1 Switchgrass plants grown in a hoop-house.

Fig. S2 Aboveground biomass in three successive years following switchgrass plant establishment in 2008.

Fig. S3 Nitrogen remobilization between switchgrass aboveground and underground organs during yearly senescence.

Table S1 Average expression of switchgrass differentially-expressed genes, with two-fold transcript change in leaves, stems and crowns (n = 3); with three-fold change in roots (n = 1) (separate Excel file)

Table S2 List of senescence-associated genes in switchgrass leaves and stems and their homologs, if available, in Arabidopsis, rice, maize and wheat (separate Excel file)

Table S3 Functional groups of switchgrass leaf and stem PvSAGs classified by MapMan (Pathways: Overview) for 426 and 321 homologous Arabidopsis loci respectively (separate Excel file)

Table S4 Expression of senescence-associated protein degradation genes (PDGs) in switchgrass leaves and stems (separate Excel file)

Table S5 List of PvSAGs encoding putative TFs in switchgrass leaves and stems and their homologs in Arabidopsis (separate Excel file)

Table S6 Coexpression clusters of PvSAGs in switchgrass leaves and stems (separate Excel file)

Table S7 The functional groups of switchgrass PvSAGs coexpressed with five target PvNACs (separate Excel file)

Table S8 Expression of upregulated genes, their Arabidopsis homologs and functional grouping in switchgrass crowns and roots during remobilization of aboveground nitrogen (separate Excel file)

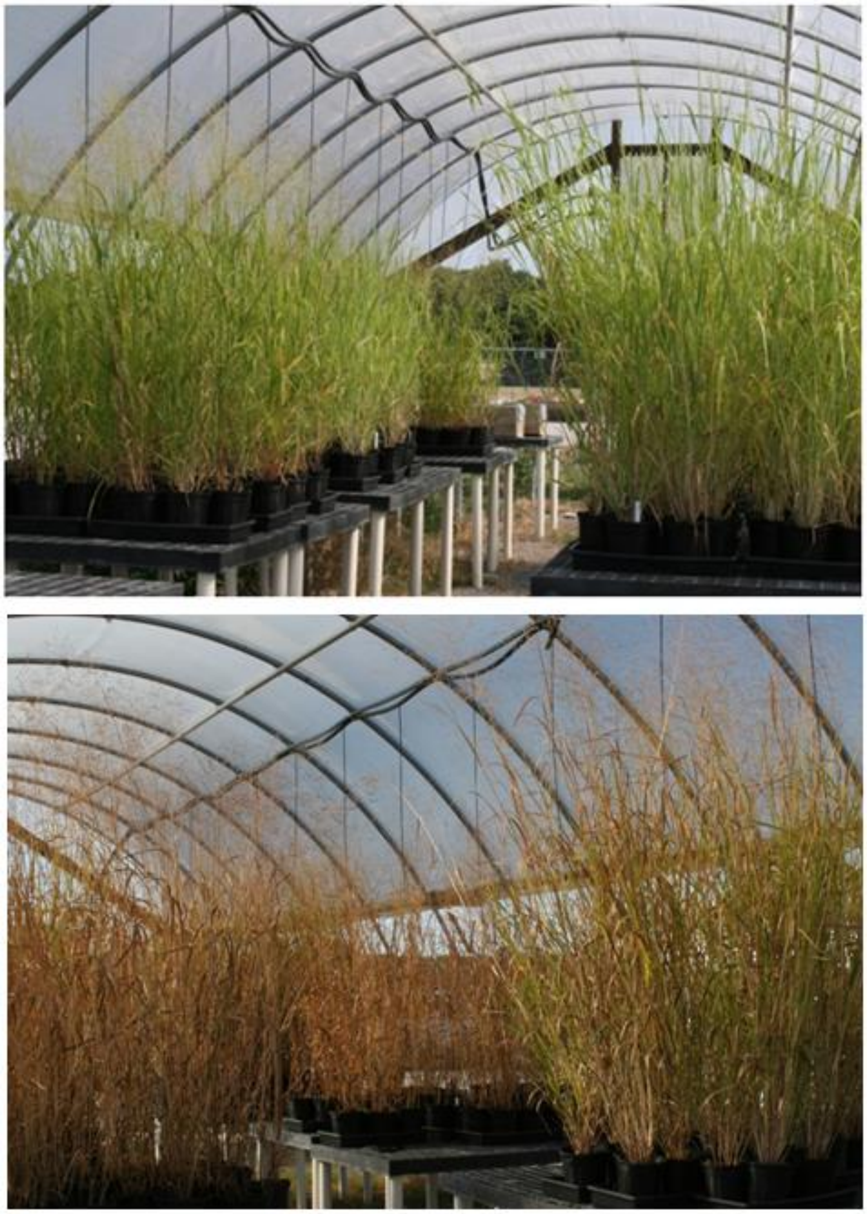

Fig. S1 Switchgrass plants grown in a hoop-house in Ardmore, OK, USA, in August (upper panel) and November (lower panel).

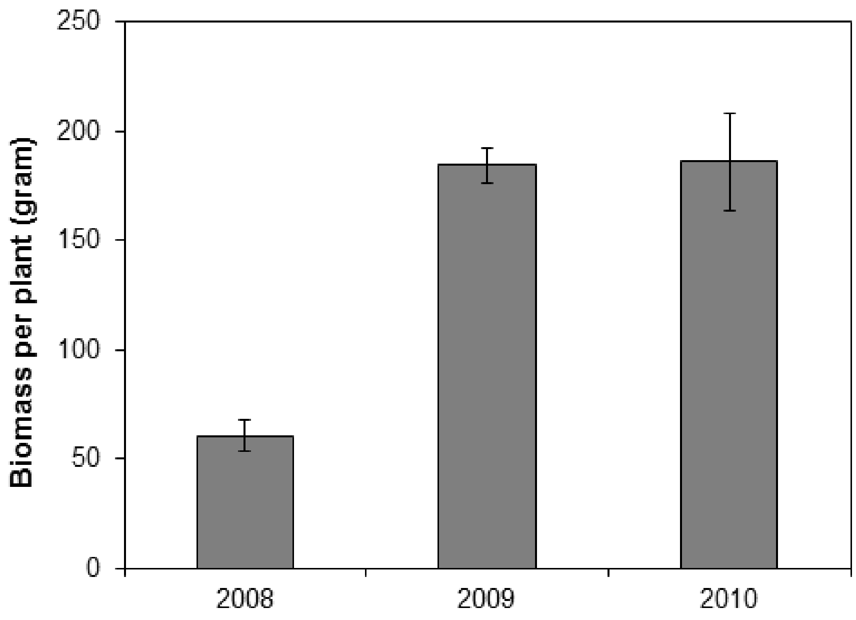

Fig. S2 Aboveground biomass in three successive years following switchgrass plant establishment in 2008. Aboveground tissue was harvested from Alamo plants in December every year. The values are mean ± SD of three replicates each of which included 45–46 plants in 2008, 30–32 plants in 2009 and 12–14 plants in 2010.

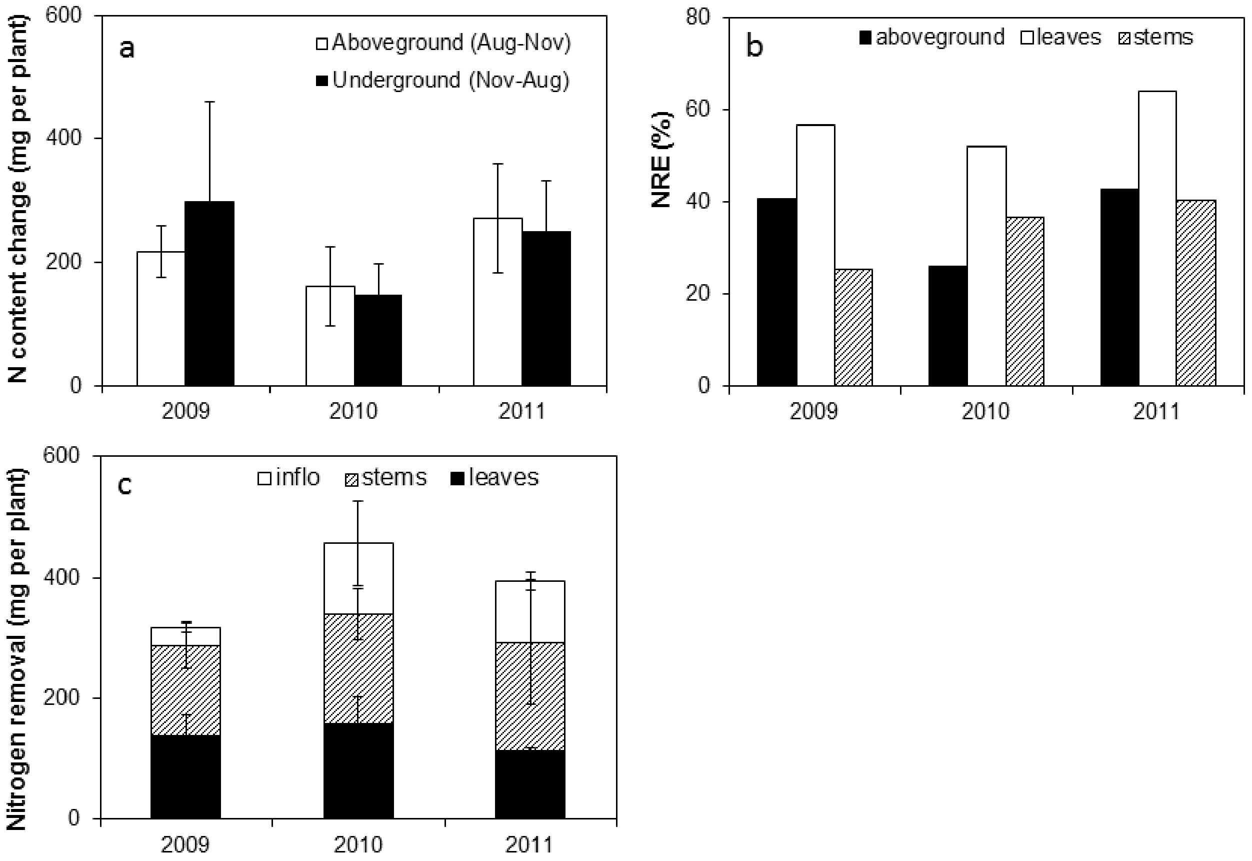

Fig. S3 (a) Nitrogen content decrease in switchgrass aboveground organs and increase in underground (determined from changes in N-content between August and November) during yearly senescence. (b) The nitrogen remobilization efficiency (NRE, %) of aboveground organs, leaves and stems estimated by ((peak N content – post-senescence N content)/peak N content) as shown in Fig. 3(c). (c) Nitrogen removal rate by specific postsenescent aboveground organs harvested in December (2009) or November (2010 and 2011).
